# Textbook of Dermatology

**Published:** 1987-05

**Authors:** R. P. Warin


### Book Reviews

### TEXTBOOK OF DERMATOLOGY

**Edited by Rook, A., Wilkinson, D. S., Ebling, F. J. G., Champion, R. H., & Burton, J. L.**
**Blackwell Scientific Publications, Oxford 1986 (4th Edition).**
**P. 2616 £180 ISBN 0–632–00949–7.**

This book was first published in 1968 by the first three listed editors with the aim of providing a comprehensive account of clinical dermatology, but introducing each chapter with an up-to-date review of the relevant basic physiopathology. The contributors were experts in each subject covered and were in the main from UK centres with a few from other parts of the world. Some of these have been replaced in the later editions. One of our Bristol dermatologists, Dr. R. R. M. Harman, with his considerable knowledge of tropical dermatology has contributed to every edition. He is the author of the sections on leprosy and also parasitic worms and protozoa.

For the fourth edition two new editors have been added so that there will be continuity when the senior editors retire. These are Dr. R. H. Champion from Cambridge and Dr. J. L. Burton from Bristol, both of whom have recently been editors of the British Journal of Dermatology. They have also contributed several chapters to the book.

After the first edition the book very quickly became established as the most authorative and comprehensive book for dermatology in the world, and it has often been referred to as the Dermatological Bible (including 'Revelations'!).

Since the first edition knowledge has increased to the extent that the text of the book has grown and it is now divided into three separate volumes, which makes its handling so much easier.

The work makes an excellent reference book and no disorder or subject seems to be left out.

In spite of its price most dermatologists and many physicians in other specialties and in general practice will want to have access to this new edition.

R. P. Warin

